# Penicillin allergy labels and antibiotic prescribing in carceral settings: expanding evidence from a statewide cohort

**DOI:** 10.1017/ash.2026.10768

**Published:** 2026-06-23

**Authors:** Kap Sum Foong, Samuel Wilk, Rachel Tam, Lindsay Taylor, Alysse Wurcel

**Affiliations:** 1 Division of Infectious Diseases, Denver Health Medical Center: Denver Health Main Campus, USA; 2 Tufts University School of Medicine, USA; 3 Boston Medical Center, USA; 4 University of Wisconsin-Madison School of Medicine and Public Health, USA

## To the Editor,

Antimicrobial stewardship efforts have historically overlooked carceral settings, despite their disproportionate burden of infectious diseases and antimicrobial exposure.^1^ Antimicrobial resistance (AMR) is amplified in these environments due to structural vulnerabilities, limited antimicrobial stewardship infrastructure, and high baseline disease burden, yet prescribing practices remain understudied.^2^ We recently reported that among incarcerated individuals receiving antibiotics across four United States (U.S.) carceral systems, penicillin allergy labels (PALs) were associated with increased risk of receiving high-risk antibiotics for *Clostridioides difficile* infection (CDI).^3^ However, that analysis was restricted to individuals already exposed to antibiotics and could not estimate population-level PAL prevalence.

To address this gap, we analyzed data from the Wisconsin Department of Corrections (DOC), leveraging a statewide census of 20,483 incarcerated individuals from 2021 to 2023. PALs were identified in 1,852 individuals (9.0%), which is comparable to prevalence estimates reported in the general U.S. population (10%).^4^ We then constructed a retrospective cohort including all individuals with a PAL and a randomly selected equal number of individuals without a PAL. Demographic characteristics and antibiotic prescriptions were collected, and antibiotics were categorized as high-risk or non–high-risk for CDI using National Healthcare Safety Network 2017 baseline criteria. Using conditional logistic regression, we evaluated associations between PAL, demographic characteristics (age, race, ethnicity), and antibiotic prescribing, including receipt of any antibiotic and high-risk antibiotics for CDI. A two-sided *P* value of <.05 was considered statistically significant. All statistical analyses were performed using SPSS, version 25 (IBM, Armonk, NY, USA). This study was reviewed by the Institutional Review Board of Tufts Medical Center and Wisconsin DOC and deemed low risk and exempt from full review.

Demographic characteristics and antibiotic classes prescribed stratified by PAL status are summarized in Supplementary Table 1. Univariable analyses of factors associated with receipt of any antibiotics and receipt of high-risk antibiotics for CDI are presented in Supplementary Tables 2 and 3, respectively. Compared with incarcerated individuals without PALs, those with PALs were more likely to be prescribed any antibiotic (adjusted odds ratio [aOR], 1.208; 95% confidence interval [CI], 1.082–1.349) and had substantially higher odds of receiving high-risk antibiotics for CDI (aOR 6.318; 95% CI, 4.837–8.253) (Table 1). Compared with our prior multistate study,^3^ the strength of association between PALs and high-risk antibiotic use was notably greater, suggesting that PALs may exert a more pronounced influence on antibiotic selection within certain carceral systems.

**Table 1.** Association of penicillin allergy labels with antibiotic prescribing outcomes in a Wisconsin DOC cohort Table 1 long description.

Variable	Receipt of any antibiotic (aOR*, 95% CI)	*P* value	Receipt of high-risk antibiotic for CDI (aOR*, 95% CI)	*P* value
Penicillin allergy label				
• No	Reference		Reference	
• Yes	1.208 (1.082–1.349)	<.001	6.318 (4.837–8.253)	<.001
Age				
• ≤ 25	Reference		Reference	
• 26–35	1.121 (0.805–1.560)	.499	2.008 (1.104–3.949)	.024
• 36–45	1.119 (0.796–1.574)	.516	1.980 (1.053–3.725)	.034
• ≥46	1.116 (0.800–1.557)	.520	2.609 (1.394–4.883)	.003
Race				
• Black	Reference		Reference	
• White	1.007 (0.849–1.194)	.938	0.898 (0.752–1.302)	.989
• Other	1.029 (0.753–1.406)	.859	1.089 (0.662–1.793)	.737
• Unknown	0.786 (0.049–12.700)	.865	Not estimable	
Ethnicity				
• Hispanic	Reference		Reference	
• Non-Hispanic	1.337 (0.853–2.096)	.205	1.111 (0.507–2.443)	.793
• Unknown	1.739 (1.121–2.696)	.013	1.199 (0.560–2.569)	.640

aOR, adjusted odds ratio; CDI, *Clostridioides difficile* infection; CI, confidence interval; DOC, Department of Corrections.

* Adjusted for age, race, ethnicity, and penicillin allergy label.

This study adds several important insights beyond our prior work. First, it provides a population-level estimate of PAL prevalence in a carceral system, rather than limiting analysis to antibiotic-exposed individuals. Second, PALs were associated not only with antibiotic receipt but also with a substantially increased likelihood of receiving high-risk agents when antibiotics were prescribed, reinforcing their role in driving broader-spectrum antibiotic use and associated CDI risk.

These findings are particularly relevant given the broader literature demonstrating that antimicrobial stewardship programs are rarely implemented in jails and prisons and that prescribing practices are highly variable across facilities.^5^ As most incarcerated individuals eventually return to the community, gaps in stewardship within carceral systems have direct implications for population-level AMR.^6^ In addition to structured allergy histories and risk stratification, direct oral challenge for low-risk patients may represent a feasible and resource-conscious stewardship intervention in carceral settings. Recent studies have demonstrated that direct oral challenge is safe, effective, less resource intensive than skin testing,^7^ and may be implemented outside of specialized allergy settings.^8^ Thus, direct oral challenge has the potential to be adopted across correctional healthcare systems with limited access to allergy specialists.

Our findings should be interpreted in the context of several limitations. Data on underlying comorbidities and chronic medical conditions were not available in the data set. Additionally, individuals without a PAL were defined by the absence of a documented PAL in the electronic medical record, which may introduce ascertainment bias if individuals prescribed antibiotics underwent more detailed allergy assessment during clinical evaluation.

In conclusion, our findings support incorporating penicillin allergy evaluation and delabeling into antimicrobial stewardship strategies in carceral settings. Targeted and resource-conscious interventions, such as structured allergy histories, risk stratification, and direct oral challenge, may represent high-yield opportunities to improve antibiotic prescribing and reduce downstream harms in this vulnerable population.

## Supporting information

10.1017/ash.2026.10768.sm001 Foong et al. supplementary material Foong et al. supplementary material

## Data Availability

The data underlying this article cannot be shared publicly due to restrictions in data use agreements but may be made available from the corresponding author upon reasonable request.

## Supplementary material

The supplementary material for this article can be found at https://doi.org/10.1017/ash.2026.10768.

## Previous presentation

This work was presented at the Infectious Diseases Society of America’s IDWeek in October 2025 in Atlanta, Georgia.

## Table 1. Long description

The table presents data on the association between penicillin allergy labels (PAL) and the receipt of antibiotics, focusing on any antibiotic and high-risk antibiotics for Clostridioides difficile infection (CDI). The table has 12 rows and 4 columns. Column headers are Variable, Receipt of any antibiotic (aOR*, 95% CI), P value, and Receipt of high-risk anti-biotic for CDI, 95% CI), P value. Row labels include Penicillin allergy label, Age, Race, and Ethnicity. Each row provides adjusted odds ratios (aOR) and 95% confidence intervals (CI) for different demographic characteristics. For example, individuals with a penicillin allergy label have an aOR of 1.208 for receiving any antibiotic and an aOR of 6.318 for receiving high-risk antibiotics for CDI, both with P values of less than 0.001. Age groups are compared with the reference group of individuals aged 25 or younger, showing varying aORs and P values. Race categories include Black, White, Other, Unknown, and Not estimable, with corresponding aORs and P values. Ethnicity categories include Hispanic, Non-Hispanic, and Unknown, with respective aORs and P values. Notable trends include higher odds of receiving antibiotics for individuals with PALs and varying odds ratios across different age groups, races, and ethnicities.

Navigate back to Table 1..
