# Supplementary material for: Penicillin allergy labels and antibiotic prescribing in carceral settings: expanding evidence from a statewide cohort
Source: Antimicrob Steward Healthc Epidemiol. 2026 Jun 23;6(1):e186. doi: 10.1017/ash.2026.10768 (PMC13312235; doi:10.1017/ash.2026.10768)
Supplement: Foong et al. supplementary material [file S2732494X26107682sup001.docx]

**Supplementary Table 1**. Demographic characteristics, antibiotic prescribing, and receipt of high-risk antibiotics for *Clostridioides difficile* infection, stratified by penicillin allergy label status

|  | Total, n=3,704 | PAL, n=1,852 | Without PAL, n=1,852 |
| --- | --- | --- | --- |
| Age Category |  |  |  |
| ≤25 | 292 | 106 | 186 |
| 26-35 | 981 | 440 | 541 |
| 36-45 | 1110 | 563 | 547 |
| ≥46 | 1321 | 743 | 578 |
| Race |  |  |  |
| Black (African American or African) | 1283 | 523 | 760 |
| White | 2148 | 1183 | 965 |
| Other | 271 | 144 | 127 |
| Unknown | 2 | 2 | 0 |
| Ethnicity |  |  |  |
| Hispanic or Latino | 127 | 57 | 70 |
| Non-Hispanic or Latino | 1100 | 548 | 552 |
| Unknown | 2477 | 1247 | 1230 |
| Total number of antibiotics prescribed (n=1375) |  |  |  |
|  |  |  |  |
| 1 | 806 | 421 | 385 |
| 2 | 343 | 192 | 151 |
| 3 | 136 | 84 | 52 |
| 4 | 54 | 39 | 15 |
| ≥5 | 36 | 20 | 16 |
| Antibiotic classes^a^ |  |  |  |
| Penicillins | 501 | 46 | 455 |
| First Gen Cephalosporin | 170 | 81 | 89 |
| Second Gen Cephalosporin | 16 | 13 | 3 |
| Third Gen Cephalosporin | 59 | 38 | 21 |
| Fourth Gen Cephalosporin | 1 | 1 | 0 |
| Fluroquinolones | 128 | 92 | 36 |
| Macrolides | 250 | 188 | 62 |
| Sulfamethoxazole-trimethoprim | 233 | 158 | 75 |
| Clindamycin | 379 | 333 | 46 |
| Tetracycline | 395 | 256 | 139 |
| Metronidazole | 145 | 93 | 52 |
| Nitrofurantoin | 38 | 29 | 9 |
| Aminoglycoside | 2 | 2 | 0 |
| Daptomycin | 1 | 1 | 0 |
| Linezolid | 2 | 2 | 0 |
| Fosfomycin | 1 | 1 | 0 |
| Dalbavancin | 1 | 0 | 1 |
| Vancomycin | 6 | 3 | 3 |
| Carbapenems | 1 | 1 | 0 |
| Receipt of high-risk antibiotics for CDI^b^, n=1375 |  |  |  |
|  |  |  |  |
| Yes | 196 | 176 | 20 |
| No | 878 | 351 | 527 |
| Both^c^ | 301 | 229 | 72 |

CDI, *Clostridioides difficile* infection**;** PAL, penicillin allergy label.

^a^Some individuals received more than 1 class of antibiotic.

^b^High-risk antibiotics for CDI were defined using NHSN 2017 baseline criteria and included third- and fourth-generation cephalosporins (cefdinir, cefepime, cefixime, cefotaxime, cefpodoxime, ceftazidime, ceftriaxone), fluoroquinolones (ciprofloxacin, gemifloxacin, levofloxacin, moxifloxacin), and clindamycin.

^c^“Both” indicates receipt of at least one high-risk and one non-high-risk antibiotic for CDI during the study period.

**Supplementary Table 2**. Univariable analysis of factors associated with receipt of any antibiotics among incarcerated individuals in Wisconsin

|  | Unadjusted OR (95% CI) | *p* value |
| --- | --- | --- |
| Age |  |  |
| ≤25 | Reference |  |
| 26-35 | 1.222 (0.885 – 1.688) | 0.223 |
| 36-45 | 1.232 (0.885 – 1.715) | 0.215 |
| ≥46 | 1.287 (0.935 – 1.771) | 0.122 |
| Race |  |  |
| Black | Reference |  |
| White | 1.090 (0.927 – 1.282) | 0.296 |
| Other | 1.080 (0.796 – 1.467) | 0.621 |
| Unknown | 1.044 (0.065 – 16.724) | 0.976 |
| Ethnicity |  |  |
| Hispanic | Reference |  |
| Non-Hispanic | 1.302 (0.834 – 2.032) | 0.246 |
| Unknown | 1.727 (1.116 – 2.672) | 0.014 |
| Penicillin allergy label |  |  |
| No | Reference |  |
| Yes | 1.221 (1.098 – 1.358) | <0.001 |

CI, confidence interval; OR, odds ratio.

**Supplementary Table 3**. Univariable analysis of factors associated with receipt of high-risk antibiotics for *Clostridioides difficile* infection among incarcerated individuals in Wisconsin

|  | Unadjusted OR (95% CI) | *p* value |
| --- | --- | --- |
| Age |  |  |
| ≤25 | Reference |  |
| 26-35 | 2.642 (1.456 – 4.791) | 0.001 |
| 36-45 | 2.634 (1.461 – 4.748) | 0.001 |
| ≥46 | 3.761 (2.103 – 6.724) | <0.001 |
| Race |  |  |
| Black | Reference |  |
| White | 1.369 (1.071 – 1.749) | 0.012 |
| Other | 1.420 (0.901 – 2.238) | 0.131 |
| Unknown | Not estimable |  |
| Ethnicity |  |  |
| Hispanic | Reference |  |
| Non-Hispanic | 1.081 (0.525 – 2.224) | 0.833 |
| Unknown | 1.225 (0.608 – 2.468) | 0.570 |
| Penicillin allergy label |  |  |
| No | Reference |  |
| Yes | 6.610 (5.078 – 8.603) | <0.001 |

CI, confidence interval; OR, odds ratio.
